# Polypoid colonic metastases from gastric stump carcinoma: A case report

**DOI:** 10.3892/ol.2014.2254

**Published:** 2014-06-16

**Authors:** BINGXIA GAO, XINYING XUE, WEIPING TAI, JINGHUI ZHANG, HONG CHANG, XIAORONG MA, YING QI, LIFANG CUI, FENGCAI YAN, LEI PAN

**Affiliations:** 1 Department of Gerontology, Beijing Shijitan Hospital, Capital Medical University, Beijing 100038, P.R. China; 2 Department of Gastroenterology, Beijing Shijitan Hospital, Capital Medical University, Beijing 100038, P.R. China; 3 Department of Pathology, Beijing Shijitan Hospital, Capital Medical University, Beijing 100038, P.R. China

**Keywords:** adenocarcinoma, signet ring cells, gastric stump cancer, metastasis, multiple colonic polyps

## Abstract

The present study aimed to investigate polypoid colonic metastases from gastric stump carcinoma by performing a retrospective analysis of the clinical data of a patient with such a diagnosis, and by discussing other previous case studies from the literature. The patient of the present study was an 80-year-old male who had undergone a gastrectomy 48 years previously for a benign perforated gastric ulcer. A colonoscopy revealed >10 multiple polypoid lesions of 6–10 mm in diameter distributed throughout the entire colon, except in the rectum. Each lesion had either erosion or a depression at the top and several were covered with a white fur-like substance. Biopsy specimens excised from the stomach showed a poorly-differentiated adenocarcinoma with diffuse signet ring cells, and a colonoscopy-guided biopsy revealed a signet ring cell adenocarcinoma. The patient was referred to the Oncology unit (Beijing Shijitan Hospital, Beijing, China) for assessment and chemotherapy treatment, which was initiated with 1,000 mg Xeloda orally administered twice a day for two-week courses every three weeks. The patient succumbed to upper gastrointestinal hemorrhage and pneumonia after three months. Gastric or gastric stump carcinoma may metastasize to the colon presenting as solitary or multiple colonic polyps. Thus, it is important to consider this diagnosis as such colon metastases may mimic solitary or multiple colonic polyps, which are commonly observed. A differential diagnosis is required in this complicated situation.

## Introduction

The dissemination of gastric neoplasms commonly occurs due to hematogenous spread, lymphatic metastases, direct local invasion of adjacent organs and peritoneal or transcoelomic spread (1). Metastases are found at the sites of the regional lymph nodes, peritoneum, liver, lungs and bones (2). The criteria for the diagnosis of metastatic tumors are well documented. Firstly, the primary tumor must be known and histologically confirmed. Secondly, the metastatic tumor must be of the same histological type as the primary tumor. Finally, the possibility of direct local spread from the primary tumor must be excluded (3). Colonic metastases are uncommon and usually originate from carcinomas of the breast, stomach, skin (melanomas), kidney, prostate, or ovaries (4). Colonic metastases from gastric adenocarcinoma usually present as ‘linitis plastica’ or as an annular stricture (5). Gastric, or gastric stump, carcinoma may metastasize to the colon and present as solitary or multiple colonic polyps, which is an extremely rare condition with <10 cases described in the literature before August 20, 2012 (www.ncbi.nlm.nih.gov/pubmed), with the first case reported by Metayer *et al* (6) in 1991, and subsequently by Ogiwara *et al* (4) in 1994. The present study reports a case of poorly-differentiated adenocarcinoma with diffuse signet ring cells of gastric stump adenocarcinoma and mucosal metastases in multiple colonic polyps. The patient provided written informed consent.

## Case report

An 80-year-old male patient who presented with the symptoms of diarrhea, weight loss, anorexia and lower abdominal pain was admitted to the Department of Geriatric Medicine (Beijing Shijitan Hospital, Beijing, China). The patient had previously undergone a gastrectomy due to the perforation of a benign gastric ulcer 48 years previously. A physical examination revealed paleness and no significant cervical or supraclavicular lymphadenopathy was noted. Breath sounds were normal and a grade 2/6 systolic apical murmur was detected upon auscultation. The laboratory examination showed a hemoglobin level of 9.9 g/dl, a lactate dehydrogenase level of 1,756 mmol/l (normal range, 40–240 mmol/l) and hydroxybutyrate dehydrogenase levels of 1,383 mmol/l (normal range, 80–200 mmol/l). The serum carcinoembryonic antigen level was 416.4 ng/ml (normal, ≤5.0 ng/ml), the carbohydrate antigen (CA)72.4 level was >300 U/ml (normal, ≤6.9 U/ml) and the CA19-9 level was 272.82 U/ml (normal, ≤37 U/ml). All other biochemical and hematological tests were normal.

**Figure 1:**
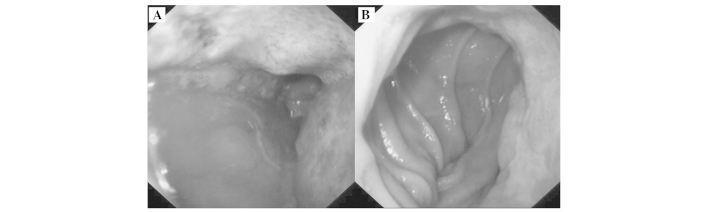
Gastroscopy images showing (A) a cardiac ulcer and (B) a gastrointestinal anastomotic ulcer.

**Figure 2:**
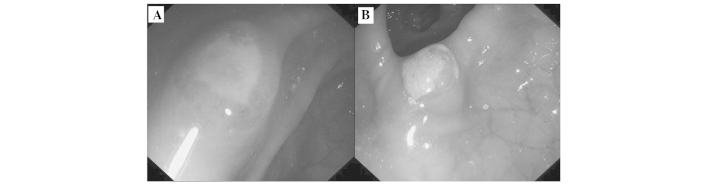
Colonoscopy images showing polypoid lesions measuring 6–10 mm in diameter in the (A) transverse and (B) descending colon. The lesions were scattered throughout the entire colon, except the rectum. Each lesion had either an erosion or a depression at the top and several were covered with a white fur-like substance .

**Figure 3:**
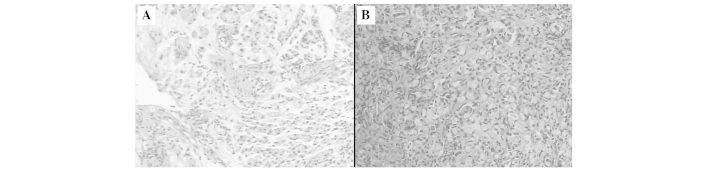
Histopathological examination results revealing (A) a poorly-differentiated adenocarcinoma with scattered signet ring cells in the stomach mucosa and (B) a signet ring cell adenocarcinoma in the colon mucosa. (Hematoxylin and eosin staining; magnification, ×100).

**Figure 4:**
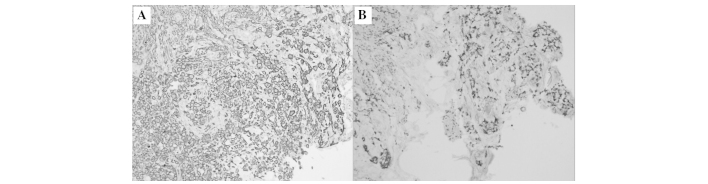
Immunohistochemical staining for CK7 and CK20 in gastric stump mucosa showing (A) CK7^+^ and (B) CK20^+^ staining (magnification, ×200). CK, cytokeratin.

**Figure 5:**
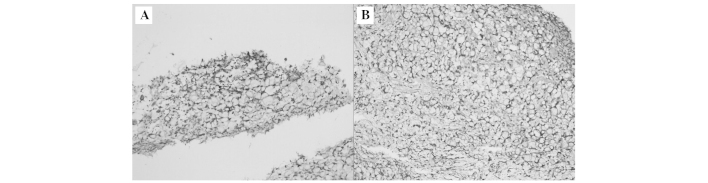
Immunohistochemical staining for CK7 and CK20 in colon mucosa showing (A) CK7^+^ and (B) CK20^+^ staining (magnification ×200). CK, cytokeratin.

Gastroscopy detected multifocal ulcerated lesions in the remnant stomach from the cardia (Fig. 1A) to the gastrointestinal anastomosis (Fig. 1B), however, the boundaries of certain lesions were unclear. Colonoscopy revealed that >10 multifocal polypoid lesions measuring 6–10 mm in diameter were scattered throughout the entire colon, except in the rectum (Fig. 2A, transverse colon; and Fig. 2B, descending colon). Each lesion had either erosion or a depression at the top, and several were covered with a white fur-like substance. Abdominal magnetic resonance imaging revealed diffuse thickening of the remnant stomach wall and multiple enlarged lymph nodes on the lesser curvature and retroperitoneum. The biopsy specimens from the stomach showed a poorly-differentiated adenocarcinoma with scattered signet ring cells (Fig. 3A), and the colonoscopy-guided biopsy revealed a signet ring cell adenocarcinoma (Fig. 3B). Immunohistochemical staining of the gastric stump mucosa (Fig. 4A and B) and colon mucosa (Fig. 5A and B) was positive for cytokeratin (CK)7 and CK20. Thus, the actual colonic lesions were corresponding with the mucosal spread of the primary gastric carcinoma.

The patient was referred to the Oncology unit for assessment, and chemotherapy consisting of 1,000 mg Xeloda was administered twice a day for one period. The patient succumbed to upper gastrointestinal hemorrhage and pneumonia after three months.

## Discussion

Gastric stump cancer occurs more frequently at the site of anastomosis, and poorly-differentiated carcinoma is the most common histological type (7). Gastric cancer spreads via several routes, including hematogenous spread, which is the most frequent mechanism by which distant metastases arise. The liver, lung and pancreas are the most common sites for gastric metastases, and direct local invasion of adjacent organs, peritoneal or trans-coelomic spread and lymphatic metastases can also occur (8). Colonic metastases from gastric cancer are extremely rare. The predominant route is known to be hematogenous, whereby metastatic deposits invade the submucosal lymphatics and extend to form a linitis plastica appearance or an annular stricture (5). The overlying mucosa may give the impression of being normal and test negative for malignancy upon mucosal biopsy, as observed in the study by Lim *et al* (9). Polypoid colonic metastases from gastric cancer have been reported in <10 cases. One such case occurred 11 years after a total gastrectomy for a poorly-differentiated adenocarcinoma of the stomach (4). A second case occurred at the colonic anastomosis, with colonic polyp mucosal metastasis of a signet ring cell gastric adenocarcinoma developing one year after a sigmoidectomy with termino-terminal anastomosis for sigmoid adenocarcinoma (2). Two cases presented with colonic metastasis at the time of the diagnosis of gastric cancer; however, yet another case was recorded by postmortem investigation (6,10–12). In the present study, the patient had undergone a partial gastrectomy for a perforated gastric ulcer 48 years previously. Polypoid colonic metastasis arising from gastric carcinoma has been recorded with the following clinical pathological characteristics: i) Poorly-differentiated cancer or differentiation of signet ring cells as the common histological type; ii) colonoscopy or barium enema revealing a solitary adenomatous colonic polyp (11–14) or polymorphic polyps (4,6,10) ranging in diameter from 2 to 15 mm, with a sessile or semi-pedunculated nature; iii) nodules scattered throughout the colon, with either erosion or a depression at the top of each; and iv) weight loss, diarrhea, melena and anorexia as the common symptoms. In addition, the primary tumor on the stomach is always a large ulcer.

In total, >96% of signet ring cell carcinoma cases originate in the stomach, with the remaining cases occurring in the colon, rectum, gallbladder, pancreas, urinary bladder and breast (15). The incidence of signet ring cell cancer in the colorectum is 0.1–2.4%, and the clinical characteristics include an advanced stage at diagnosis, a large tumor size, a proximal location, a young patient age, a propensity for lymphovascular invasion and peritoneal seeding (16).

As colon signet ring cell adenocarcinomas are rare, the differential diagnosis of a primary colon or metastatic gastric cancer is debated when a signet ring cell carcinoma is diagnosed via colonoscopy. Immunohistochemical analyses are performed to differentiate between a gastric and colonic primary tumor, with CK7 and CK20 commonly used as tumor markers. CK7 expression has been observed in the majority of carcinoma cases, with the exception of those cases in which the cancers originated from the prostate, colon, thymus and kidney, in carcinoid tumors originating from the lungs and gastrointestinal tract and in Merkel cell tumors of the skin. CK20-positive staining has been found in almost all colorectal carcinoma and Merkel cell tumor cases, as well as a high percentage of patients with pancreatic carcinoma (62%), gastric carcinoma (50%), cholangiocarcinoma (43%) and transitional cell carcinoma (29%). It has been hypothesized that when a signet ring cell adenocarcinoma is revealed on colon biopsy, the diagnosis of a colonic origin is supported by the presence of a CK7^−^/CK20^+^ staining pattern in the neoplastic cells, while a gastric origin is diagnosed when the cells have a CK7^+^/CK20 staining pattern (15). However, Chu *et al* (18) reported that 13% (1/8) of cases of gastric carcinomas and 5% (1/20) of colorectal carcinomas were CK7^+^/CK20^+^. In addition, Wang *et al* (19) reported that 38% (11/29) of gastric adenocarcinomas and 10% (4/40) of colorectal adenocarcinomas were CK7^+^/CK20^+^; thus, CK7^+^/CK20^+^ staining pattern is more common in gastric adenocarcinomas than in colorectal cancer. In the present case, the biopsy specimens were positively stained for CK7 and CK20. The colonic lesions were multifocal, therefore the actual colonic lesions corresponded with the mucosal spread of the primary gastric cancer. A previous study has hypothesized that tissues of chronic inflammation may provide a spectrum of mitogen and trophic signals that make this area more favorable for the establishment of tumor metastasis (2). However, the routes by which lymphatic or hematogenous metastases occur could not be excluded in the present study. There were certain limitations to the study, as an endoscopic ultrasound was not performed for colonic lesions, therefore the source of the lesions was not found.

In conclusion, gastric or gastric stump carcinoma may metastasize to the colon and present as solitary or multiple colonic polyps. This carcinoma is an extremely rare condition with <10 cases described in the literature up until August 20, 2012 (www.ncbi.nlm.nih.gov/pubmed). Therefore, it is important to consider gastric carcinoma as a possible diagnosis, as colon metastases may mimic solitary or multiple colonic polyps, which are more commonly observed. In such complicated cases, a differential diagnosis is required.
